# Post-processing radio-frequency signal based on deep learning method for ultrasonic microbubble imaging

**DOI:** 10.1186/s12938-019-0714-6

**Published:** 2019-09-11

**Authors:** Meng Dai, Shuying Li, Yuanyuan Wang, Qi Zhang, Jinhua Yu

**Affiliations:** 1 Department of Electronic Engineering, Fudan University, Shanghai, 200433 China; 2 Key Laboratory of Medical Imaging Computing and Computer Assisted Intervention of Shanghai, Shanghai, 200433 China; 3 School of Communication and Information Engineering, Shanghai University, Shanghai, 200444 China

**Keywords:** Microbubble, Ultrasound contrast agent, Radio frequency (RF) signal, U-net, Eigenspace, Ultrasound contrast agent plane wave imaging

## Abstract

**Background:**

Improving imaging quality is a fundamental problem in ultrasound contrast agent imaging (UCAI) research. Plane wave imaging (PWI) has been deemed as a potential method for UCAI due to its’ high frame rate and low mechanical index. High frame rate can improve the temporal resolution of UCAI. Meanwhile, low mechanical index is essential to UCAI since microbubbles can be easily broken under high mechanical index conditions. However, the clinical practice of ultrasound contrast agent plane wave imaging (UCPWI) is still limited by poor imaging quality for lack of transmit focus. The purpose of this study was to propose and validate a new post-processing method that combined with deep learning to improve the imaging quality of UCPWI. The proposed method consists of three stages: (1) first, a deep learning approach based on U-net was trained to differentiate the microbubble and tissue radio frequency (RF) signals; (2) then, to eliminate the remaining tissue RF signals, the bubble approximated wavelet transform (BAWT) combined with maximum eigenvalue threshold was employed. BAWT can enhance the UCA area brightness, and eigenvalue threshold can be set to eliminate the interference areas due to the large difference of maximum eigenvalue between UCA and tissue areas; (3) finally, the accurate microbubble imaging were obtained through eigenspace-based minimum variance (ESBMV).

**Results:**

The proposed method was validated by both phantom and in vivo rabbit experiment results. Compared with UCPWI based on delay and sum (DAS), the imaging contrast-to-tissue ratio (CTR) and contrast-to-noise ratio (CNR) was improved by 21.3 dB and 10.4 dB in the phantom experiment, and the corresponding improvements were 22.3 dB and 42.8 dB in the rabbit experiment.

**Conclusions:**

Our method illustrates superior imaging performance and high reproducibility, and thus is promising in improving the contrast image quality and the clinical value of UCPWI.

## Background

Ultrasound contrast agents (UCAs) [1] enable ultrasound diagnosis to discover small lesions and have triggered a new round of technical innovation in the ultrasound imaging [2–4]. UCA for clinical use are usually microbubbles whose mean diameter is less than a red blood corpuscle. The microbubble is inert-gas-filled and encased by a shell to stabilize it and prevent the dissolution. After entering the body by intravenous injection, UCA can enhance the ultrasonic backscattering intensity and image contrast, resulting in the improvement of visual effect of imaging and the accuracy of clinical diagnosis.

With further development, ultrasound contrast agent imaging (UCAI) has become more widely used in clinical diagnosis. Meanwhile, conditions such as low mechanical index which are essential to UCAI have been highly emphasized in clinical examination [5, 6] since microbubbles can be easily broken under high mechanical index conditions. Plane wave imaging (PWI), due to its’ several advantages, has been deemed as a potential method for UCAI and attracted a lot of attention [7, 8]. The high frame rate of PWI makes it possible to track fast moving microbubbles. And the low mechanical index of PWI can reduce the disruption of microbubbles to a large extent. However, the clinical practice of ultrasound contrast agent plane wave imaging (UCPWI) is still limited by poor image quality for lack of transmit focus. Over the past 25 years, many methods [9–18] have been applied to improve UCPWI and shown promising results. These methods enhance the contrast between the microbubbles and other tissues by utilizing the nonlinear characteristics of microbubbles [9, 10]. Pulse inversion [11], amplitude modulation [12], chirp-encoded excitation [13], golay-encoded excitation [14], second harmonic imaging [15], sub-harmonic imaging [16], super-harmonic imaging [17] and bubble approximated wavelet transform (BAWT) [18] are the representatives of methods that have significant effect. Most of these methods improve the imaging contrast-to-tissue ratio (CTR) based on the time–frequency difference between microbubbles and tissues. In most cases, the tissues only produce linear echoes while the harmonic components are contributed by microbubbles. Although it is feasible to distinguish tissues and microbubbles according to their spectral difference, when the mechanical index beyond some level, tissues will also produce harmonic signals due to the nonlinear distortion of waveforms, and the spectrum aliasing between the microbubbles and tissues will become an unfavorable factor [19]. Our previous work [20] used a bubble area detection method to improve the image quality; the outstanding performance showed that removing the tissue signal interferences is a promising research direction for UCPWI improvement. However, when facing strong scattering points, the previous work still showed its deficiencies in the recognition of tissue signals.

To identify ultrasound radio frequency (RF) signals from different areas effectively, we introduced deep learning [21], which offers excellent classification capability. As an important branch of machine learning, deep learning allows computational models to dig out high-throughput features from huge amount of data. The continuous improvement of computer hardware in recent years has enabled deep learning to make full use of its advantages and made it become a non-negligible choice for medical data analysis. Generally, the application of deep learning includes four parts: the data set, the network structure, the cost function and the optimization algorithm [22]. In the last century, the achievement of the Convolutional Neural Network (CNN) in the field of face recognition has attracted widespread attention [23]. CNN is one of the most widely used algorithms in deep learning and has been successfully applied in computer vision, speech recognition, and medical image analysis [24, 25]. Recurrent neural network (RNN) is another commonly used network, which is particularly advantageous for the processing of sequential data [26]. Different from the traditional neural network structure, each node of the RNN is connected. The RNN has a memory of the historical input data. U-net network was proposed in 2015 [27]. Based on CNN, U-net added the upsampling layer for deconvolution operation. The combination of the convolutional layer and the pooling layer is equivalent to a quadratic feature extraction structure. This structure empowers the network consider the deep and the shallow features simultaneously, and thus it can improve the effectiveness of the network.

In this study, we extended our previous work [20] and proposed a new post-processing method for UCPWI, Table 1 shows the key differences between the previous method and the proposed. The proposed method consists of three stages: (1) First, we applied the idea of deep learning to trained a model based on U-net, which can effectively identify tissue signal interferences. (2) Then BAWT combined with maximum eigenvalue threshold was employed to eliminate the remaining tissue RF signals. (3) Finally, the accurate microbubble image was obtained through eigenspace-based minimum variance (ESBMV) imaging algorithm. Both phantom and rabbit in vivo experiments were performed to validate the proposed method. The experimental results showed the proposed method has a great potential in advancing the ultrasound diagnosis of contrast imaging.

**Table 1:** Key differences between the previous methods and the proposed method

Method	The previous method	The proposed method
Strength	Simpler, no need for large amounts of data	More accurate bubble area prediction with trained model
Weakness	Unable to accurately predict the location of bubble area	Need to collect a lot of data to train the network

## Result

The U-net network was based on the keras deep learning framework and the TITAN Xp GPU was used for computing acceleration. It took about 25 min for one iteration. The subsequent beamforming algorithm was applied using matlab.

The training and testing accuracy of the three networks was up to 0.95 and the area of the receiver operating characteristic curve (ROC) was higher than 0.9, indicating that the networks have good prediction and generalization capabilities.

### Phantom experiment results

First, to select the network structure and the beamforming algorithm that best meet the needs, we discussed the classification ability of the three network structures and imaging performance of the three beamforming algorithms. And then we compared the results when the three network algorithms combined with the three beamforming algorithms, respectively, based on CTR and contrast-to-noise ratio (CNR) values. The expression of the CTR and CNR can be described as follows:

1 $${\text{CTR}} = 20\log \frac{{I_{\text{UCA}} }}{{I_{\text{tissue}} }}$$

2 $${\text{CNR}} = 20\log \frac{{I_{\text{UCA}} - I_{\text{tissue}} }}{{\sqrt {\sigma_{\text{UCA}}^{2} + \sigma_{\text{tissue}}^{2} } }}$$

where $I_{\text{UCA}}$ and $I_{\text{tissue}}$ are the mean intensity of contrast and tissue, $\sigma_{\text{UCA}}$ and $\sigma_{\text{tissue}}$ are the corresponding standard deviation. Finally, the influences of BAWT and maximum eigenvalue threshold were discussed.

**Fig. 1:**
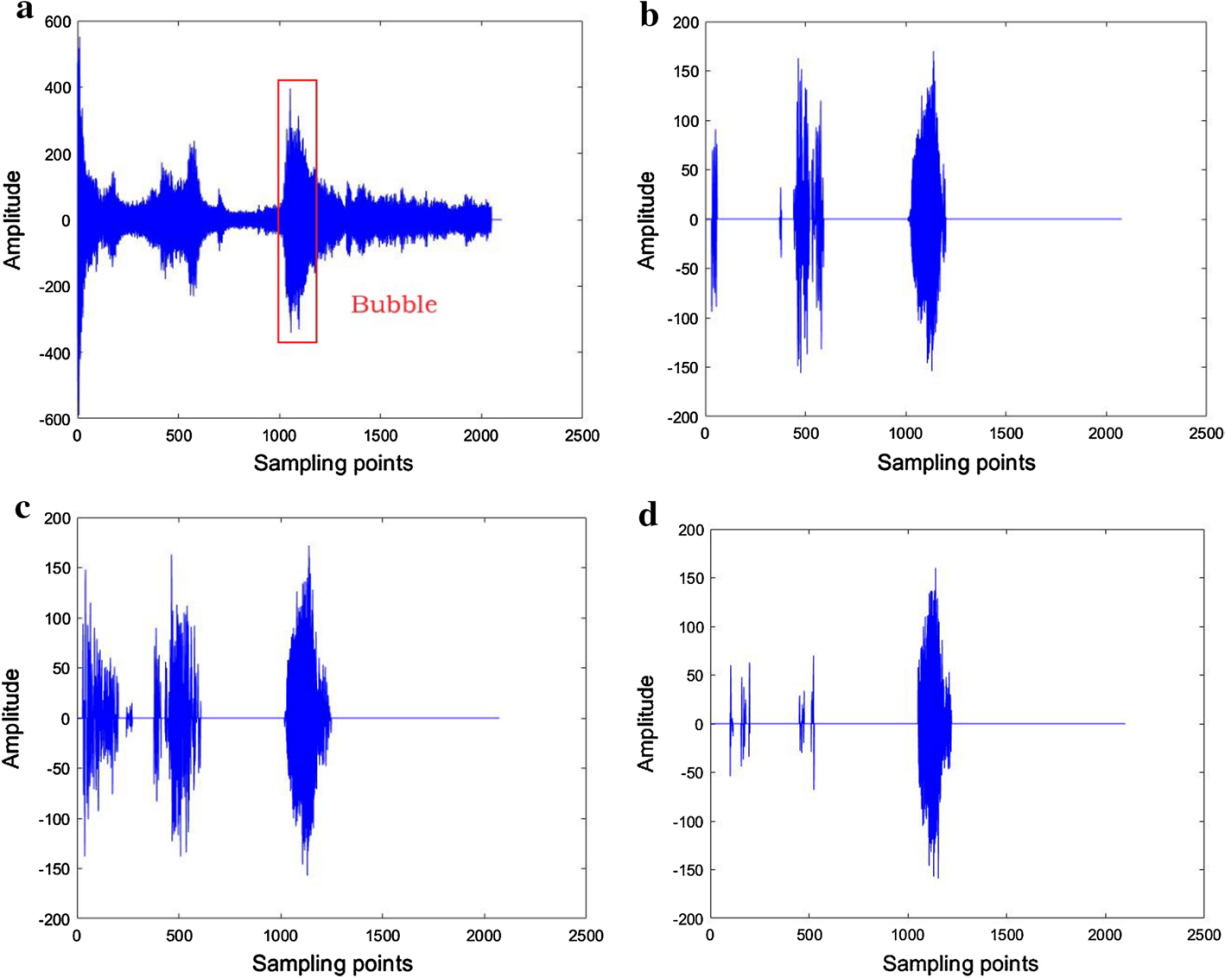
The RF signal waveform before and after classification. **a** Before classification, **b** after CNN classification, **c** after RNN classification, **d** after U-net classification

Figure 1 gives a comparison of the RF signal waveforms before and after deep learning classification. Based on the distance and the size of the phantom, the rectangular box in Fig. 1a denotes the microbubble areas, and the front part corresponding to the pork interfaces. In the original RF signal, the amplitudes of the pork signal and the microbubble signal have little difference. After classification with deep learning network, the ranges of RF signals from microbubbles can be located easily. From experiment, it can be observed that the strong interferences from pork tissues have been reduced effectively by U-net, and partially by CNN and RNN.

**Fig. 2:**
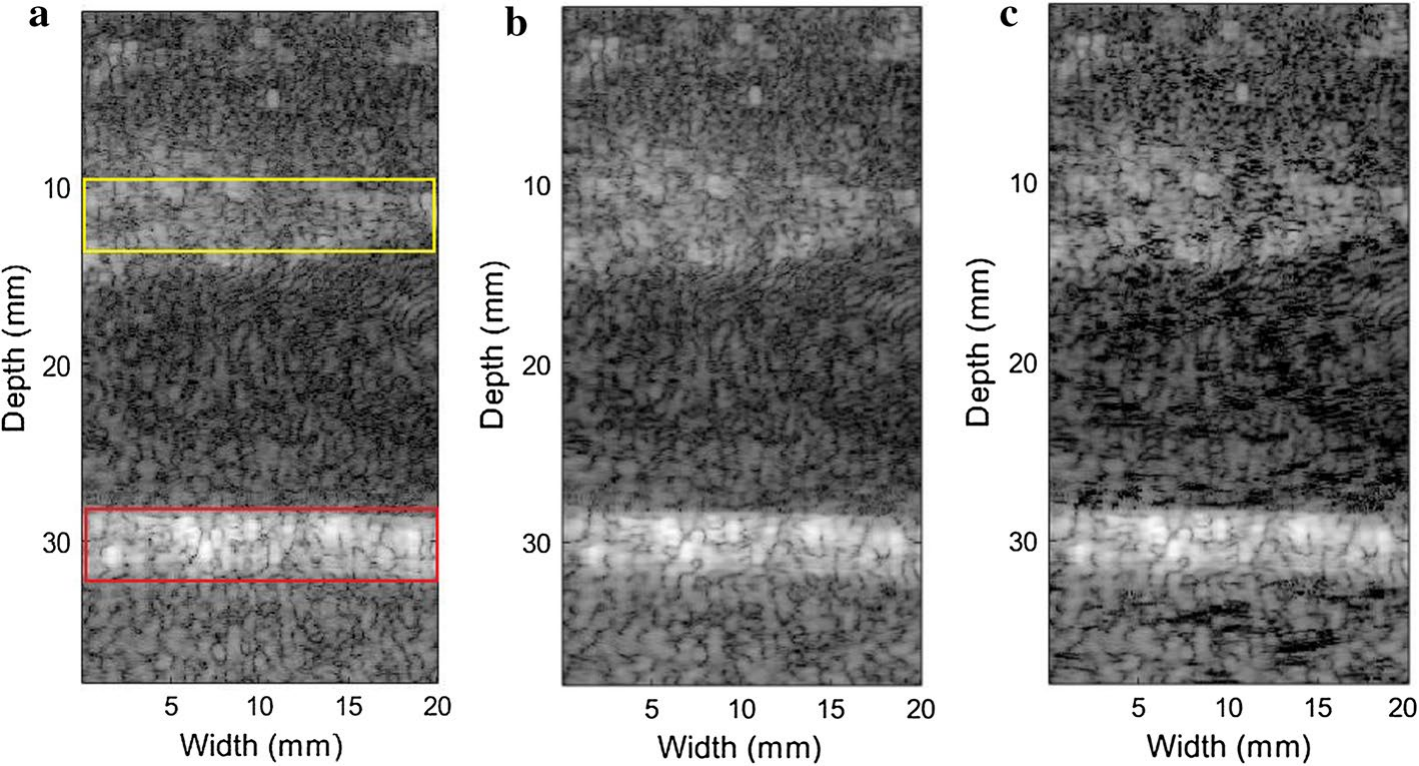
The image result of the pork phantom experiment (the yellow rectangle in Fig. 5a is the tissue area and the red one is the microbubble area). **a** Traditional DAS, **b** traditional MV, **c** traditional ESBMV

Figure 2 are the traditional DAS, MV, and ESBMV beamforming imaging results (the yellow rectangle in Fig. 2a is the tissue areas and the red one is the microbubble areas). There are strong scattering points in the pork signals.

Table 2 shows the CTR and CNR values when the three network algorithms combined with the three beamforming algorithms, respectively.

**Table 2:** The CTR and CNR of the pork phantom experiment

Method	CTR (dB)	CNR (dB)
Original DAS	− 7.5	− 13.4
Original MV	− 9.4	− 11.5
Original ESBMV	− 9.6	− 11.2
CNN + DAS	− 22.1	− 4.9
CNN + MV	− 23.3	− 4.5
CNN + ESBMV	− 25.7	− 3.8
RNN + DAS	− 17.2	− 6.1
RNN + MV	− 18.8	− 5.6
RNN + ESBMV	− 20.7	− 4.9
U-net + DAS	− 22.8	− 4.5
U-net + MV	− 24.2	− 3.8
U-net + ESBMV	− 26.3	− 3.5

Among the three network structures, the effect of U-net is significant, and best meets our expectations. Among the three beamforming algorithms, ESBMV is better than DAS and MV.

**Fig. 3:**
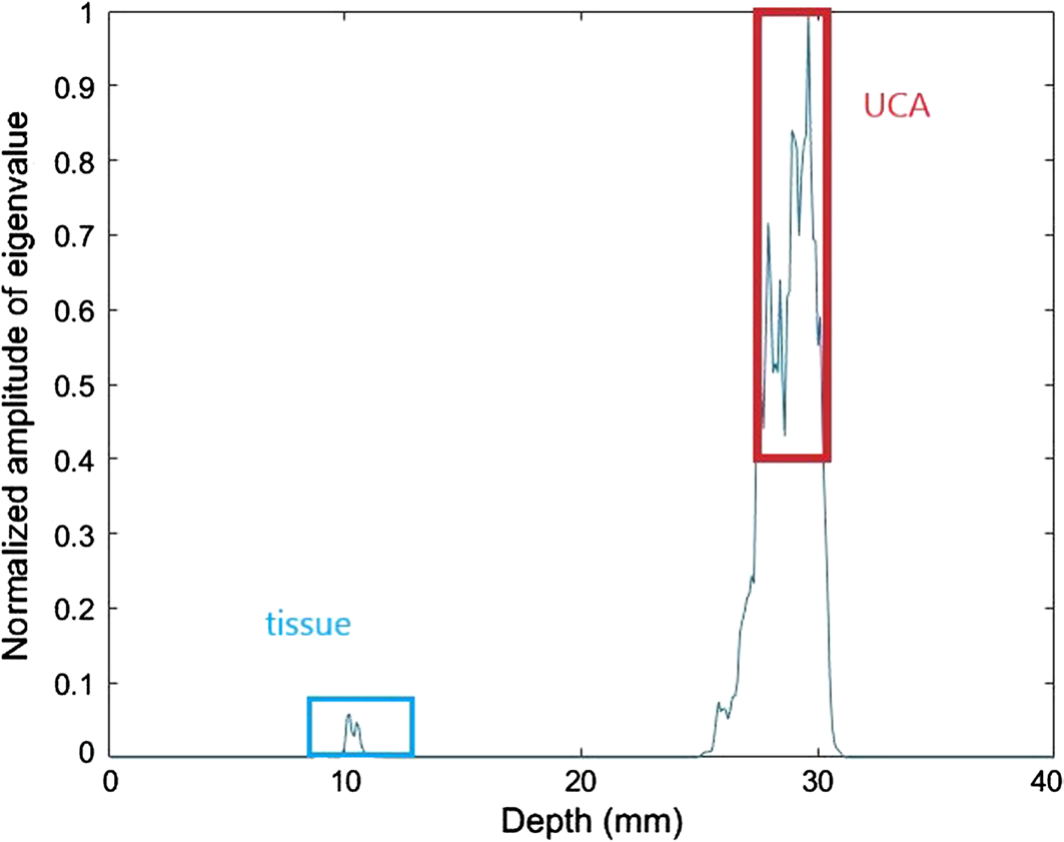
The maximum eigenvalue curve of different depths. The red rectangle represents the UCA area. The blue rectangle represents the tissue area

Then we get rid of the residual tissue signals by utilizing the maximum eigenvalue of each imaging point. Taking the area at the width of 10 mm as an example, the maximum eigenvalue curve under different depths is shown in Fig. 3. The area in the red rectangle represents the microbubble area and the blue one represents the tissue area. Its maximum eigenvalue is quite larger than other areas due to the existence of strong scattering signals produced by the microbubble. Hence, we can eliminate the pork section by setting an eigenvalue threshold.

**Fig. 4:**
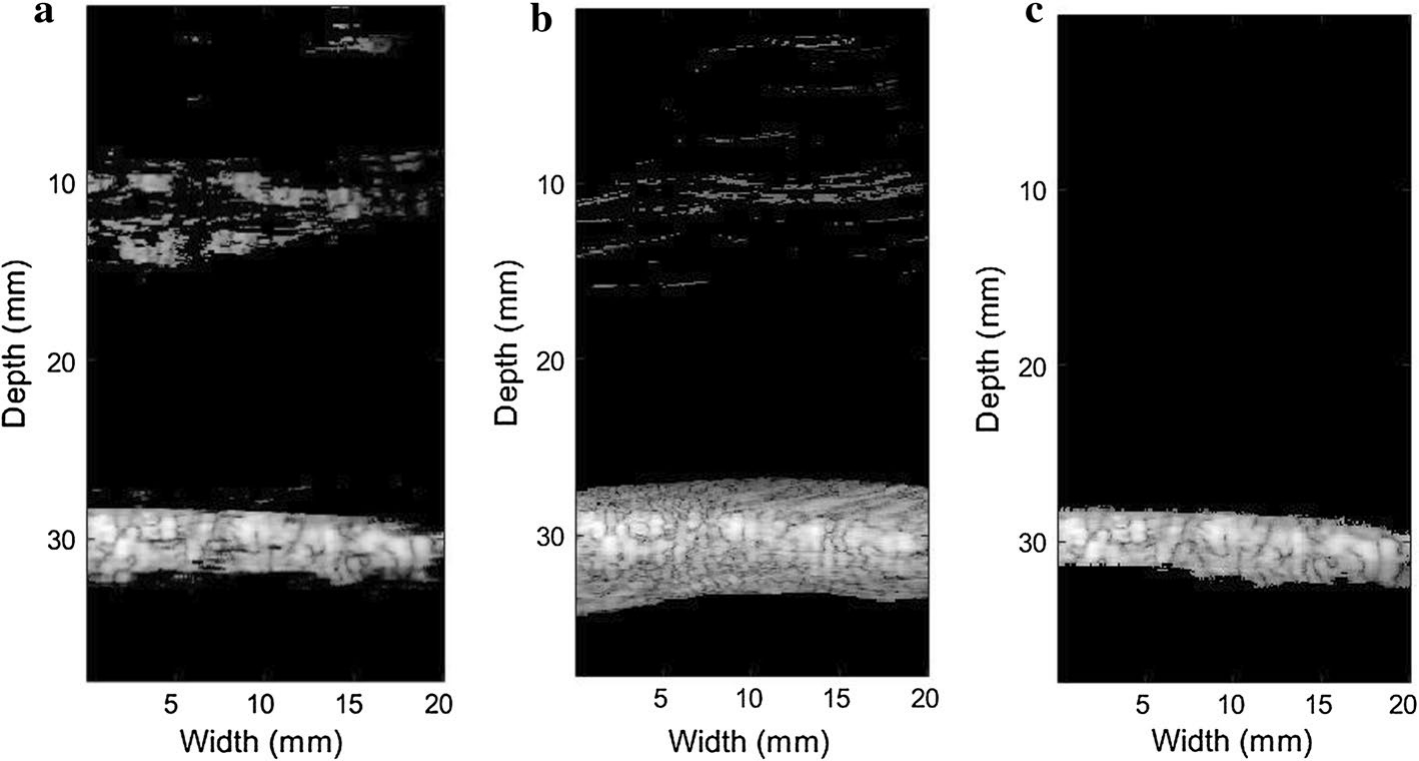
The image result of the pork phantom experiment. **a** BAWT combined with maximum eigenvalue threshold (without deep learning), **b** ESBMV after U-net classification with BAWT, **c** the proposed method (Utilizing BAWT combined with maximum eigenvalue threshold)

Besides, the microbubble area brightness can be enhanced by BAWT. Figure 4 shows the results of the proposed method and when BAWT combined with maximum eigenvalue threshold was directly implemented without deep learning. For Fig. 4a, deep learning is not involved, and the performance is unsatisfactory when facing strong scattering points. For Fig. 4c, with deep learning, the proposed method can completely eliminates the pork information, including the strong scattering point which is difficult to remove, and the degree of retention of microbubble information is high. Figure 4b is the result after deep learning classification. Notably, compared with Fig. 4a, large artifacts appeared near the boundary of the microbubble area as shown in Fig. 4b. In other words, the deep learning method has a slightly weak effect on the classification of the areas near the microbubbles. After eigenvalue threshold was set, the final result in Fig. 4c shows that artifact interferences near the boundary of the microbubble area have been reduced to a large extent.

Table 3 compares the CTR and CNR values when different methods implemented. As seen from the table, by utilizing BAWT combined with maximum eigenvalue threshold, the proposed method produced better CTR and CNR, and is more in line with our expectations.

**Table 3:** The CTR and CNR of the pork phantom experiment

Method	CTR (dB)	CNR (dB)
Original DAS	− 7.5	− 13.4
Original MV	− 9.4	− 11.5
Original ESBMV	− 9.6	− 11.2
BAWT combined with maximum eigenvalue threshold (without deep learning)	− 17.9	− 6.0
U-net + ESBMV	− 26.3	− 3.6
U-net + ESBMV + BAWT	− 27.9	− 3.3
U-net + ESBMV + BAWT + Eigenvalue threshold (the proposed method)	− 28.8	− 3.0

### In vivo experiment results

**Fig. 5:**
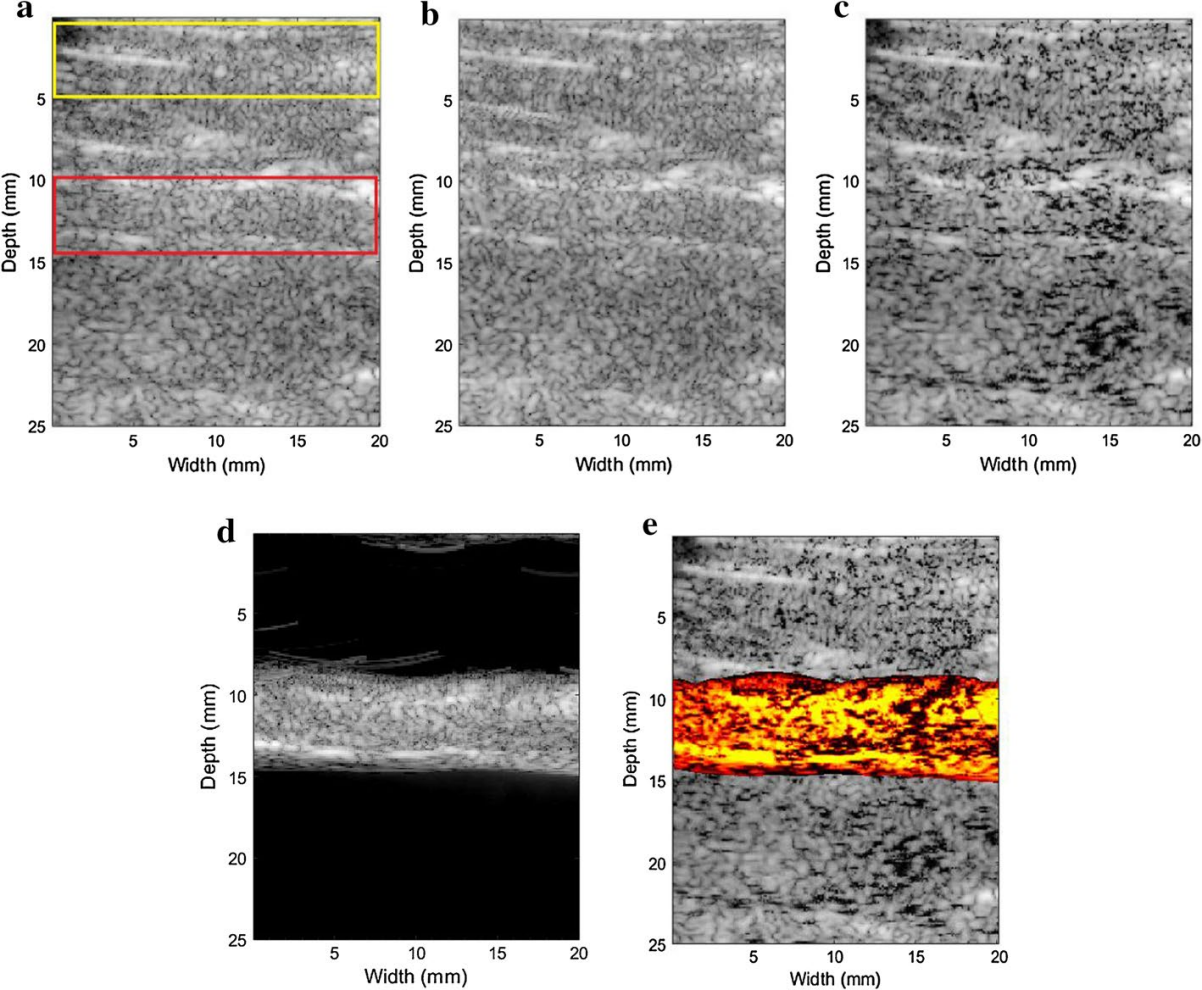
The in vivo rabbit abdominal artery result. **a** DAS, **b** MV, **c** ESBMV, **d** ESBMV + deep learning, **e** the proposed method (the yellow rectangle in Fig. 8a is the tissue area and the red one is the microbubble area)

Figure 5 shows the rabbit abdominal artery imaging results. Figure 5a–c are the original images based on different beamforming algorithms. For Fig. 5a, the yellow rectangle is the tissue area and the red one is the microbubble area. The quality of the original image is very poor and the contrast area is submerged in the background noise. Figure 5d is ESBMV-based imaging result after using deep learning to classify RF signals. Deep learning weakens tissue signals to some extent. Figure 5e shows the result of the proposed method, the detected microbubble area is displayed in color to facilitate the actual observation.

The CTR and CNR of different beamforming algorithms are shown in Table 4.

**Table 4:** The image CTR and CNR of in vivo rabbit experiment

method	CTR (dB)	CNR (dB)
DAS	− 0.27	− 47.2
MV	− 0.48	− 44.6
ESBMV	− 0.97	− 37.9
U-net + ESBMV	− 13.8	− 8.1
Proposed method	− 22.6	− 4.4

### Parameter choosing experiment results

Finally, to discuss the effect of iteration numbers, batch samples, and the length of the segmentation signals for the U-net, we also carried out many experiments. As was shown in Table 5, the network parameters have a certain influence on the deep learning classification results. In all of our experiments, the optimal signal length is 60, iteration is 150 and batch size is 100. When the deep learning is combined with the eigenvalue, the final imaging results have a small difference.

**Table 5:** The result under different network parameters of the phantom experiment

Signal length + iterations + batch sizes	Proposed method CTR (dB)	Proposed method CNR (dB)
45 + 150 + 100	− 28.2	− 3.1
50 + 150 + 100	− 28.3	− 3.1
55 + 150 + 100	− 28.5	− 3.1
60 + 150 + 100	− 28.8	− 3.0
65 + 150 + 100	− 28.6	− 3.1
60 + 100 + 100	− 28.7	− 3.0
60 + 50 + 100	− 28.2	− 3.1
60 + 150 + 50	− 28.3	− 3.1
60 + 150 + 150	− 28.6	− 3.0

## Discussion

In this paper, a novel approach was presented to improve the quality of contrast-enhanced ultrasound imaging by combining deep learning approach, BAWT and maximum eigenvalue threshold. Our work provides three main contributions: (1) A three-stage post-processing method has been proposed to improve UCPWI; (2) To the best of our knowledge, we are the first one to apply deep learning approach to improve the imaging quality of UCPWI; (3) The performance of the three network structures in tissue and microbubble RF signals classification were discussed. By considering the RF signal as a one-dimensional signal, the identification between tissue and microbubble RF signals was achieved with deep learning approach. A large number of RF signals were collected through experiments to construct a data set. The signals were processed by the U-net network, and the microbubble RF signals were located. Then BAWT combined with maximum eigenvalue threshold was used to eliminate the remaining tissue RF signals and enhance the brightness of the microbubble area. Finally, the accurate microbubble imaging was obtained through ESBMV. Both phantom and in vivo rabbit experiment results showed different degrees of improvements in the quality of contrast-enhanced ultrasound imaging.

With the help of large training data sets and its learning ability, deep learning showed excellent performance in reducing most of the tissue signals. To reduce the residual interference areas, BAWT and maximum eigenvalue threshold was applied. BAWT can enhance the UCA area brightness, and eigenvalue threshold can be set to eliminate the interference area due to the large difference of maximum eigenvalue between UCA and other areas. Compared the improvements in different stages, most of the interference areas were reduced by the deep learning method, the role of BAWT and eigenvalue threshold is to further remove interference areas near the boundary. However, even the performance of the proposed method was mainly contributed by the deep learning method, the assistant of BAWT and eigenvalue threshold is still necessary to get the accurate location information of UCA area.

The proposed method has showed superior imaging performance in advancing the quality of UCPWI. The improvements in the phantom experiments and the in vivo experiments also suggested the proposed method has good robustness and adapts to different application scenarios. And with higher hardware environment, the proposed method can maintain the advantage of fast imaging speed. Therefore, the proposed method can be a general strategy in the clinical diagnosis of UCPWI to quickly obtain the location information of blood vessels or other target areas that can be influenced by contrast agent. In practice, an overall consideration is also suggested, after using the proposed method to quickly obtain the location information of the UCA area, the original image may be referred to confirm the boundary information and reduce the uncertainties.

There are some impact factors and limitations of the proposed method. The training data sets have a great impact on the performance of deep learning; richer data sets can make the network capture more features and perform better. The proposed method improved UCPWI by increasing the computational complexity, and thus the computing speed should be guaranteed by a higher hardware environment. Considering the large scale improvement of image quality and the development of hardware environments are inevitable, to increase the computational complexity to improve UCPWI is still a worthwhile measure.

## Conclusion

The purpose of this study was to propose and validate a new post-processing method that combined with deep learning to improve the imaging quality of UCPWI. The proposed method consists of three stages: (1) First, with large training data sets, a deep learning model based on U-net was trained to differentiate microbubble and tissue radio frequency (RF) signals; (2) Then, to eliminate the remaining tissue RF signals, BAWT combined with maximum eigenvalue threshold was employed, BAWT can enhance the UCA area brightness, and eigenvalue threshold can be set to eliminate the interference areas due to the large difference of maximum eigenvalue between UCA and other areas; (3) Finally, the accurate microbubble imaging were obtained through ESBMV. Both phantom and in vivo rabbit experiments results validated the improvements. Compared with UCPWI based on DAS, the CTR and CNR was improved by 21.3 dB and 10.4 dB in the phantom experiment, and 22.3 dB and 42.8 dB in the in vivo experiment. The proposed method showed that the deep learning can contribute to highlight the UCA area and can be regarded as a general strategy to improve the performance of UCPWI. In further study, we can concentrate on developing more appropriate network to enhance the difference between UCA and tissue area, especially the distinction in the border area near the microbubble area. At the same time, the training data sets have a great impact on the performance of deep learning, we will continue to collect standard and enrich the data sets in the future.

## Materials and method

### Deep learning network structure

**Fig. 6:**
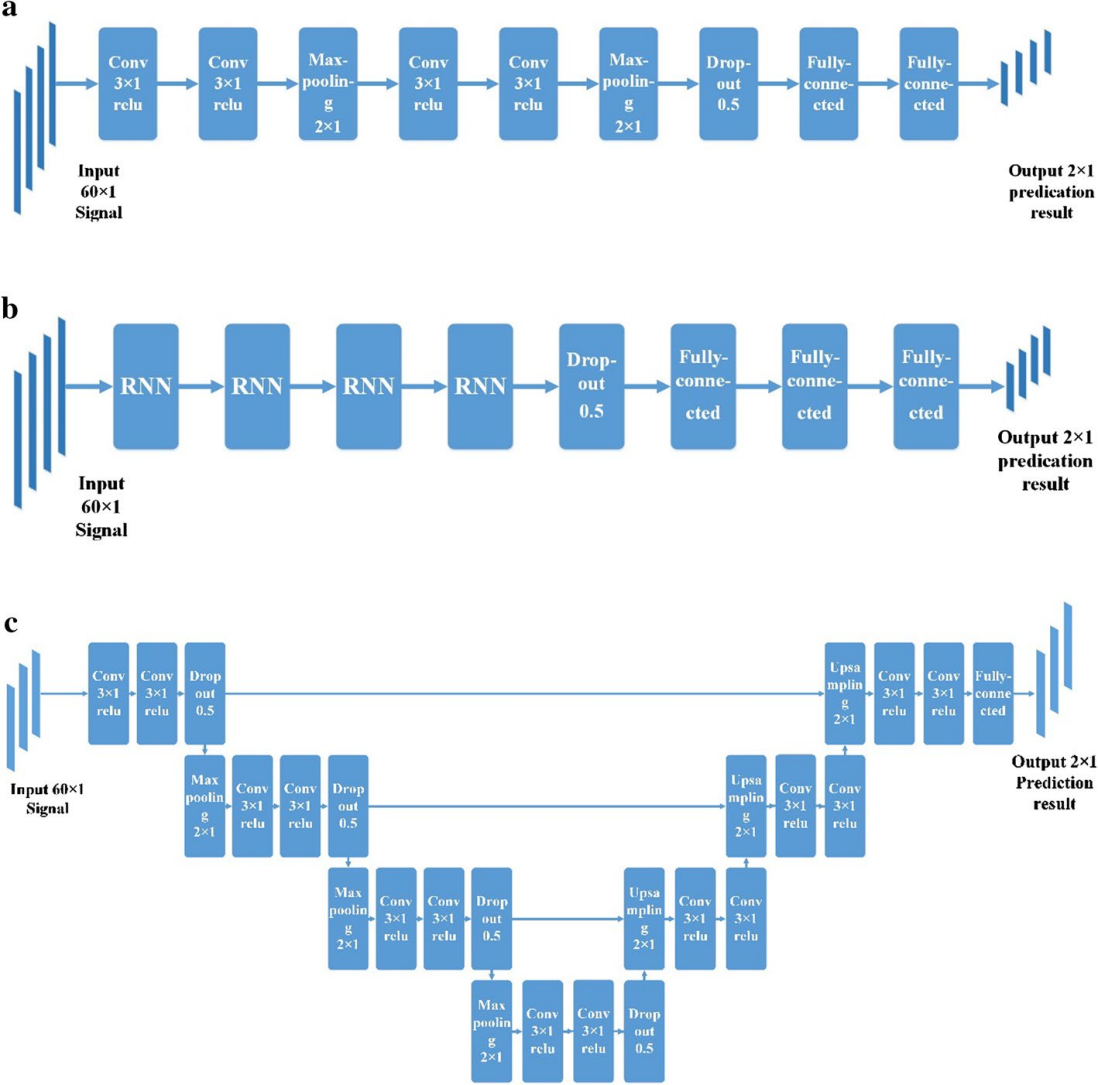
The structure of the three networks. **a** CNN, **b** RNN, **c** U-net

Three deep learning networks (including CNN, RNN, and U-net) were designed to analyze the RF signals of UCPWI. The network extracted the internal complex structure of the input data to obtain high-level data representation. The structures of the three networks are shown in Fig. 6. Network with the best experimental results was adopted in the proposed method.

The structure of the CNN network is two convolution layers with 128 filters, a layer of maximum pooling, two convolution layers with 64 filters, a maximum pooling layer, one dropout layer, and two fully connected layers.

The structure of the RNN including four RNN layers with 100 neurons, one dropout layer, and three fully connected layers. The RNN layer can take into account the information between each segment of the input signals. The output of RNN is not only related to the current input, but also the input at the previous moment.

The hidden layer of U-net consists of three upsampling layers, three downsampling layers, four dropout layers, fourteen convolutional layers, a fully connected layer, and three fusion layers. The network has a total of 607,112 parameters. The input layer was a set of one-dimensional RF signal with a length of 60, followed by a combination of convolution layer + active layer + dropout layer + downsampling layer, for a total of three times. The shallow convolution layer extracted the simpler features while the deeper convolution layer extracted more advanced and complex features. The number of the filters increased with the depth of the network. And the number of obtained feature maps increased by 32, 64, and 128 in order. Following the downsampling layer was a deconvolution step, where the number of filters decreased with the increase of the network depth, and the size of the feature map increased. Each deconvolution feature map was connected with the corresponding convolutional feature map. After that was a fully connected layer.

The convolutional layer was used to extract the signal characteristics. The size of the convolutional filter in CNN and U-net structure was chosen to be 3 × 1 with a step size of 1. In actual processing, we performed zero-padding on the edges of the data so that the size of the data obtained after the convolution process was constant. The nonlinear activation function we used after each convolutional layer was the rectified linear unit function (ReLU) [28]. Compared to the most commonly used sigmoid functions [29] in previous years, ReLU can accelerate the convergence of network. The downsampling layer used the maximum pooling with a size of 2 × 1, which means that the maximum value of this 2 × 1 window is retained and the resulting feature map size is halved. The downsampling layer was used to reduce the feature dimensions and extract some of the most important features.

The dropout layer was a commonly used method to suppress overfitting [30]. The fully connected layer combined the extracted local features into global features. After the fully connected layer, the softmax activation function was used to obtain the probability of each signal belongs to these two categories. The cost function we used was cross-entropy.

The optimization algorithm we used was Adam [31], which can adjust the learning rate adaptively to update the weights. The Adam algorithm has four hyper parameters: (1) the step-size factor, which determines the update rate of the weight the smaller the step, the easier it is for the network to converge, but the training time will be longer. (2) Epsilon, which is usually a small constant, to prevent the denominator from being zero. (3) Beta1 controls the exponential decay rate of the first moment of the gradient; (4) Beta2 controls the exponential decay rate of the second moment of the gradient.

Table 6 shows the parameter values of the three networks.

**Table 6:** The network parameter value

Parameter name	Parameter value	Parameter name	Parameter value
Batch size	100	Epoch	150
The length of the signal	60	Step-size factor	1 × 10^−5^
Beta1	0.9	Beta2	0.999
Epsilon	1 × 10^−8^	Dropout rate	0.5

### Bubble approximated wavelet transform and eigenvalue threshold

By identifying the microbubble RF signals with deep learning, we can reduce interferences from other tissues specifically. However, the microbubble signals detected by deep learning tend to contain small portion of tissue signals, which will degrade the image quality due to the intensity disparity between microbubble and tissue signals. To remove the remaining tissue signals and further improve the contrast imaging quality, BAWT combined with eigenvalue method was employed.

BAWT is a new type of post-processing technology for contrast imaging, which improves the imaging CTR while retaining the advantages of low-energy and high-frame-rate of PWI. First, the microbubble scattering sound pressure obtained by simulating the microbubble model was used as a new mother wavelet [18]. Then the continuous wavelet transform was performed on the RF signal and obtained a series of wavelet coefficients which had the same scale as the original RF signal.

In the time domain, BAWT represents the convolution operation of the processed signal and the mother wavelet at different scale factors, describing their correlation. Since the microbubble signal has a greater correlation with the mother wavelet, the resulting wavelet coefficient is larger. In contrast, the correlation between the tissue signal and the mother wavelet is relatively low, and the corresponding wavelet coefficient is small. Therefore, BAWT can further suppress the tissue signals to a certain extent, enhance the microbubble signals, and result in the improvement of the imaging CTR. The selection of the mother wavelet was based on the high-matched spectrum between the mother wavelet and the actual bubble echo. The scale factor changes the center frequency of the passband of the bubble approximated wavelet. The optimal scale factor should be chosen at whose center frequency falls at the second harmonics of the microbubbles [20].

The bubble approximated wavelet was constructed based on Doinikov model [32], which has been proven to predict the ‘compression-only’ behavior of Sonovue very well. The Doinikov model can be described as

3 $$\begin {aligned} \rho_{l} \left( {RR^{''} + \frac{3}{2}R^{'2} } \right) & = \left( {p_{0} + \frac{{2\sigma (R_{0} )}}{{R_{0} }}} \right)\left( {\frac{{R_{0} }}{R}} \right)^{3\gamma } - \frac{{2\sigma (R_{0} )}}{R} - 4\chi \left( {\frac{1}{{R_{0} }} - \frac{1}{R}} \right) \\ & \quad - P_{0} - P_{\text{drive}} (t) - 4\eta_{l} \frac{{R^{'} }}{R} - 4\left( {\frac{{k_{0} }}{{1 + \alpha \left| {\frac{R'}{R}} \right|}} + \kappa_{1} \frac{{R^{'} }}{R}} \right)\frac{{R^{'} }}{{R^{2} }} \end {aligned}$$

where *ρ*_*l*_ = 10 00 kg/m^3^ denotes the density of the surrounding liquid. *P*_0_  = 101,000 Pa as the atmospheric pressure. *γ * = 1.07 as the gas thermal insulation coefficient. *R*_0_ = 1.7 μm as the initial radius of microbubble. *R* is the instantaneous radius of microbubble. *R′* is the first-order time derivative of *R*, with essentially *R′ * = d*R*/d*t* and *R″ * = d^*2*^*R*/d*t*^*2*^. *σ*(*R*_0_) = 0.072 N/m as the initial surface tension. *χ * = 0.25 N/m as the shell elasticity modulus. *ŋ*_*l*_ = 0.002 PaS as the liquid viscosity coefficient. *k*_0_ = 4e−8 kg and *k*_1_ = 7e−15 kg/s as the shell viscosity components. *α * = 4 μs as a characteristic time constant. *P*_drive_(*t*) is the driving ultrasound.

The pressure scattered by the microbubble can be expressed as

4 $$P(d) = \rho_{l} \frac{R}{d}\left( {2R^{'2} + RR^{''} } \right)$$

where *d* denotes the distance from the center of the microbubble to the transducer.

Following this, the bubble approximated wavelet can be obtained by solving Eqs. (3) and (4) based on ODE solver provided by Matlab with the initial condition of *R*(*t* = 0)  = * R*_0_, *R′*(*t* =  *0*)= 0. The solver solves the second-order ordinary differential equation by Runge–Kutta method.

It has been proved that the eigenvalue has the ability to distinguish the microbubble and tissue area [20]. Based on the observation of the experiments, we found that the amplitude of the maximum eigenvalue in the UCA area is obviously higher than the tissue area.

The eigenvalues can be calculated as follows.

Assuming that the delayed array signal is *x*^*d*^(*k*). The array signals were divided into multiple sub-arrays of the same length and the average of the sample covariance of all sub-arrays was used as the final covariance matrix

5 $$R(k) = \frac{1}{M - L + 1}\sum\limits_{p = 1}^{M - L + 1} {x_{d}^{p} } (k)x_{d}^{p} (k)^{\text{H}}$$

where *M* is the array number of the probe. *M* − *L *+ *1* is the number of overlapping subarrays. *L* is the length of the subarray. (*·*)^H^ is the conjugate transpose. *p* is the subarray number.

Diagonal loading technology was introduced to improve the stability of the algorithm, which is

6 $$\tilde{R} = R + \varepsilon I,\;\varepsilon = \delta *{\text{trace}}(R)$$

where *I* represents the identity matrix. trace(*R*) is the main diagonal element sum of *R*. $\delta$ is a constant not greater than 1/*L*.

Next, the covariance matrix was decomposed and the eigenvalues were sorted. The signal subspace was composed of the eigenvectors corresponding to the larger eigenvalues and the eigenvectors corresponding to the smaller eigenvalues constructed the noise subspace as

7 $$R = U\varLambda U^{\text{H}} = U_{\text{S}} \varLambda_{\text{S}} U_{\text{S}}^{\text{H}} + U_{\text{P}} \varLambda_{\text{P}} U_{\text{P}}^{\text{H}} = R_{\text{S}} + R_{\text{P}}$$

where $\varLambda \, = \,{\text{diag}}[\lambda_{1} ,\lambda_{2} , \ldots \lambda_{L} ]$ are the eigenvalues in descending order. *U *= [*V*_*1*_,*V*_*2*_,*…V*_*L*_] is the eigenvector matrix. *V*_*i*_ is the eigenvector corresponding to *λ*_*i*_. *R*_S_ is the signal subspace. *R*_*P*_ is the noise subspace. *N* is used to decompose *R* into the signal subspace *U*_*s*_= [*U*_*1*_,*U*_*2*_,*…U*_*N*_] and noise subspace *U*_*P*_= [*U*_*N*+*1*_,*U*_*N*+*2*_,*…U*_*L*_]. In general, *λ*_*N*_ is set to be smaller than *λ*_*1*_ *α* times or larger than *λ*_*L*_ *β* times.

### ESBMV beamformer

The final image was obtained through the beamforming algorithm. The beamforming algorithm is a key component of ultrasound imaging and plays an extremely important role in improving the imaging quality. The beamforming algorithm improves the image quality by adaptively weighting each image point of the received array signal. delay and sum (DAS) is the most common algorithm. The echo signals received by different array elements are delayed and summed. Since each imaging point has a fixed weight, its resolution and contrast are low, and the image quality is poor. The minimum variance (MV) algorithm [33] starts the development of the adaptive beamforming. It can flexibly assign different weights to each imaging point according to the characteristics of the echo signal. MV calculates the weight by minimizing the output energy and can effectively improve the image resolution. Since the improvement of the contrast of MV is not significant, the eigenspace-based minimum variance [34] algorithm was proposed. ESBMV decomposes the array signal into two mutually orthogonal signal subspaces and noise subspaces based on the eigenvalues, and then projects the MV weights to the decomposed signal subspaces, thereby improving the imaging contrast.

The ESBMV was calculated as follows.

1. MV minimizes the array output energy
  8 $${ \hbox{min} }w^{\text{H}} Rw,{\text{ subject to }}w^{\text{H}} \;d = 1$$
  where *R* is the covariance matrix of the delayed signal. *w* is the weight vector. *d* is the direction vector.
2. Calculate the MV weight
  9 $$W_{\text{MV}} = \frac{{R^{ - 1} d}}{{d^{\text{H}} R^{ - 1} d}}$$
3. The final MV output is
  10 $$S_{\text{MV}} (k) = \frac{1}{M - L + 1}\sum\limits_{{{\text{p}} = 1}}^{M - L + 1} {W_{\text{MV}}^{\text{H}} } x_{d}^{p} (k)$$
4. Calculate the signal covariance matrix according to Eq. (5) and decompose the covariance matrix according to Eq. (7).
  The ESBMV weight can be expressed as
  11 $$W_{\text{ESBMV}} = U_{\text{S}} U_{\text{S}}^{\text{H}} W_{\text{MV}}$$
5. Finally, the ESBMV output is
  12 $$S_{\text{ESBMV}} (k) = \frac{1}{M - L + 1}\sum\limits_{{{\text{p}} = 1}}^{M - L + 1} {W_{\text{ESBMV}}^{\text{H}} } x_{d}^{p} (k)$$

### Implementation of the proposed method

**Fig. 7:**
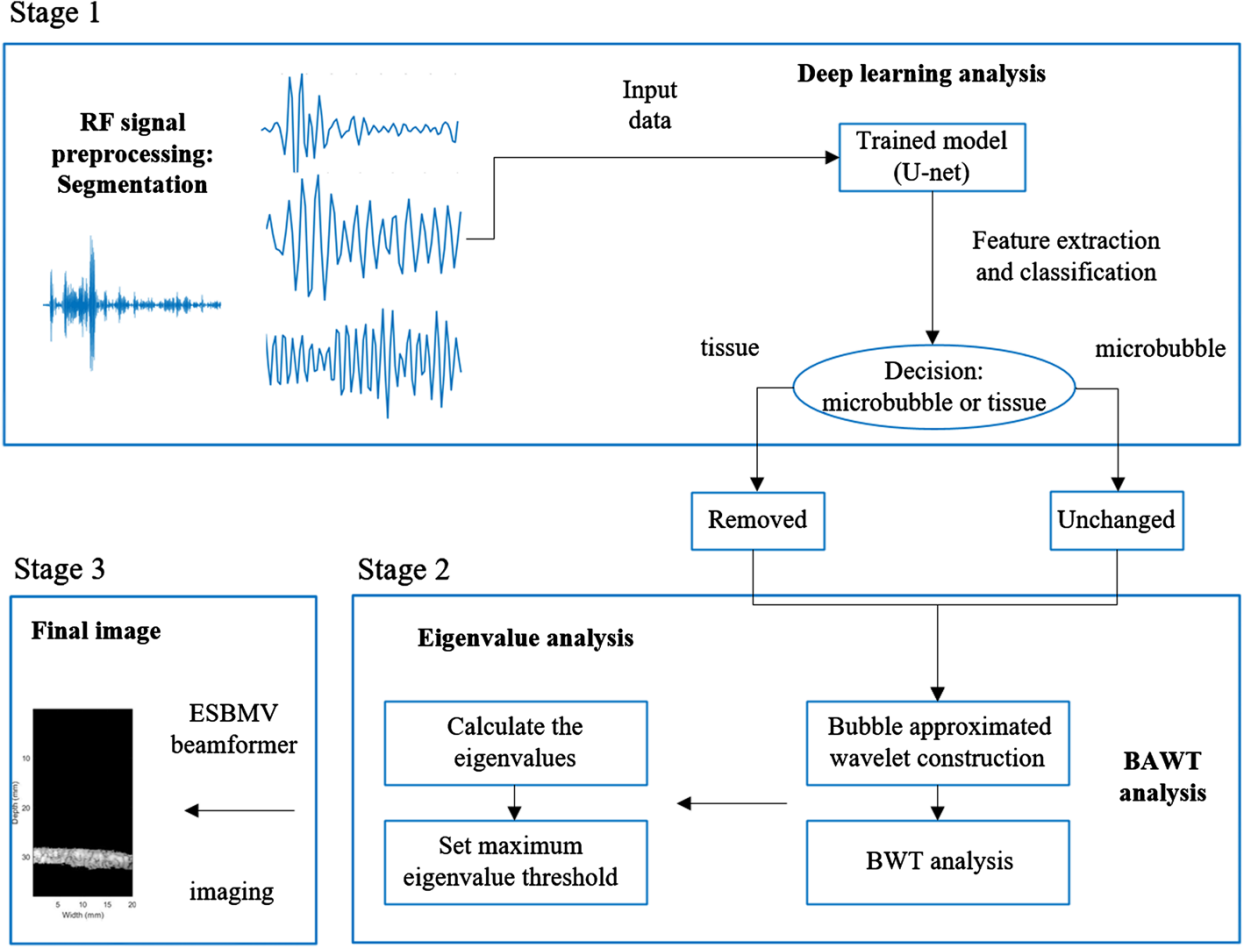
The algorithm flow

Figure 7 is the schematic view of the proposed method.

The entire algorithm flow is as follows:

1. The original RF signal was classified by U-net and the microbubble area was roughly located.
2. BAWT was used to enhance the signal of the microbubble area, and the classified RF signal was replaced with the wavelet coefficient under the optimal scale factor.
3. The signal covariance matrix was calculated according to Eq. (5) and decomposed according to Eq. (7) (*L *= 32, *α *= 0.4).
4. Based on the previous steps, the maximum eigenvalue of each imaging point were obtained.
5. The maximum eigenvalue threshold was set to determine whether it is a microbubble area (*c* times larger than the maximum eigenvalue of each scan line, *c *= 0.15).
6. For the microbubble area, the ESBMV output was calculated according to Eq. (12).
7. The final image was obtained after envelope detection and logarithmic compression (dynamic range: 60 dB).

### The collection of data set

The experimental platform was designed based on an ultrasonic research platform Verasonics Vantage 128 (Verasonics, Inc., Kirkland, WA, USA), a linear array transducer (L11-4v), four homemade gelatin phantoms, a medical syringe, a computer, Sonovue microbubble (Bracco Suisse SA, Switzerland), four pieces of fresh pork and three female rabbits (4 months, 2 kg). All animal experiments were performed according to protocols approved by Fudan University Institutional Animal Care and Use Committee.

Verasonics was used to excite the ultrasound wave and collect the RF data. The microbubble signal samples were echo signals scattered from microbubble area, including the microbubble solution in the beaker, the microbubble echoes in the phantom and the microbubble echoes in rabbit carotid artery; the tissue signal samples were echo signals scattered from tissue area, including the pork signals, gelatin phantom signals, rabbit kidney signals, rabbit carotid artery signals and rabbit belly arterial signals. To enrich the data, we changed the experimental parameters (such as the transmit frequency, the transmit voltage, the concentration of the gelatin used to make the phantom, the location and size of the internal tube of the phantom, the microbubble concentration).

Phantom (with pork) and rabbit abdominal artery experiments were used for independent testing. The phantom was made of gelatin with a wall-less tube whose diameter was 3 mm (11 cm in length, 11 cm in width, 6 cm in height). The fresh pork (taken from the belly) was used to simulate the complexity of biological tissue. For the phantom experiment, we placed a piece of fresh pork (12 mm in thickness, 40 mm in length, and 25 mm in width) over the phantom. The ultrasonic coupling gel was applied between the pork and the phantom to ensure the signal transmission. The flowing Sonovue solution (diluted by 1000 times with 0.9% physiological saline) was injected into the tube by a medical syringe. For the rabbit experiment, the rabbit was first anesthetized and then placed on an autopsy table where the four limbs were fixed by ropes. Before imaging, the area of interest was epilated to remove the influence of cony hair. Medical ultrasonic coupling gel was applied to the area of interest. A total of 500 μL Sonovue microbubbles (no dilution) were injected through the right ear vein, which was followed by 500 μL of physiological saline.

**Fig. 8:**
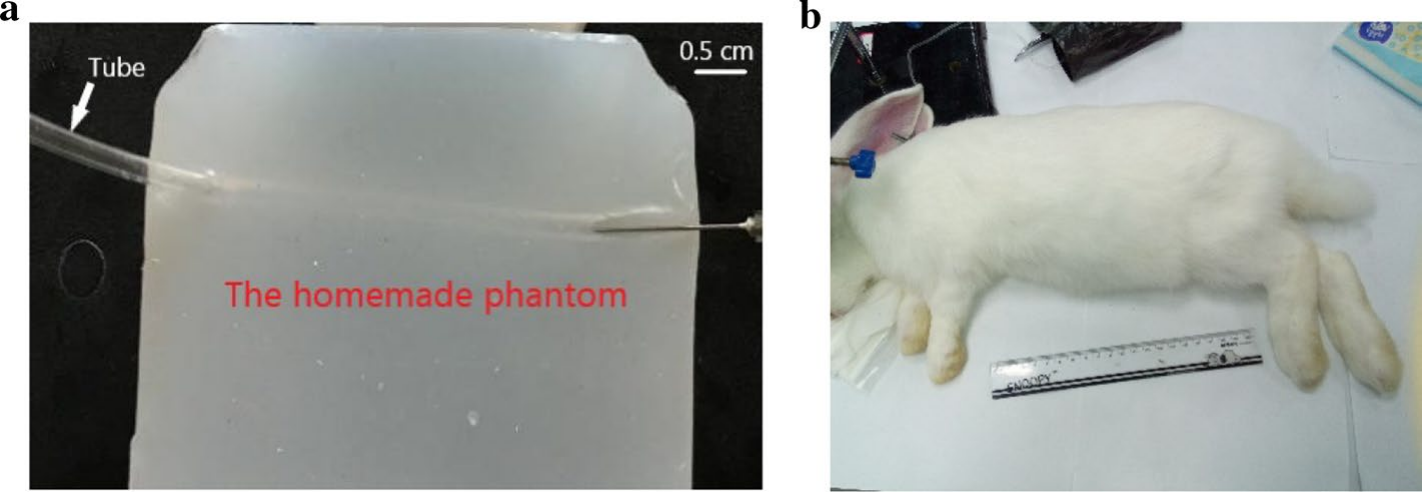
The experiment photos. **a** The phantom made of gelatin with a wall-less tube whose diameter was 3 mm (11 cm in length, 11 cm in width, 6 cm in height). **b** In vivo rabbit, the region of interest was epilated to remove the influence of cony hair before imaging, medical ultrasonic coupling gel was applied to the region of interest. A total of 500 μL Sonovue microbubbles (no dilution) were injected through the right ear vein, which was followed by 500 μL of physiological saline

Figure 8a, b shows the homemade phantom and the rabbit experiment targeting the kidney, respectively.

Table 7 gives the detailed parameters of the ultrasound instrument for the independent testing and cross validation experiment. The mechanical index was less than 0.1. The bandwidth of the probe is 4–11 MHz.

**Table 7:** Parameters of the ultrasound instrument for the experiment

Experiment parameters	Value (independent testing)	Value (cross validation)
Transducer element number	128	128
Transducer element kerf	0.05 mm	0.05 mm
Transducer element width	0.27 mm	0.27 mm
Transducer element pitch	0.3 mm	0.3 mm
Transducer spacing between elements	0. 295 mm	0. 295 mm
Transmit frequency	4 MHz	2.5 MHz, 3 MHz, 4 MHz, 5 MHz, 6.25 MHz
Transmit voltage	10 V	1.6 V, 2.5 V, 5 V, 7.5 V, 10 V, 12.5 V, 15 V, 17.5 V, 20 V
Transmit pulse	Sine wave with two cycles	Sine wave with two, three cycles
Sampling rate	25 MHz	25 MHz

The RF signal collected by Versonics have a dimension of 2100 × 128, where 128 was the number of element channels and 2100 was the length of the signal on each scan line. The RF signals (time domain) on each scan line were processed in segments, with a step size of five sampling points. The length of signal is 60 in each segment and these segments are taken as data samples to train the network.

The total number of the collected data samples is 8,694,572, of which the microbubble signal samples account for 45% and the tissue signal samples account for 55%. Such huge data sets can meet our requirement. The data were randomly divided into a training set and a test set, the training set accounted for 80% and the test set accounted for 20%.

## Data Availability

The datasets used and/or analyzed during the current study are available from the corresponding author upon reasonable request.

## Abbreviations

UCAI: ultrasound contrast agent imaging
UCAs: ultrasound contrast agents
PWI: plane wave imaging
RF: radio frequency
BAWT: bubble approximated wavelet transform
DAS: delay and sum
MV: minimum variance
ESBMV: eigenspace based minimum variance
CTR: contrast-to-tissue ratio
CNR: contrast-to-noise ratio
UCAs: ultrasound contrast agents
ReLU: rectified linear unit function
CNN: Convolutional Neural Network
RNN: recurrent neural network
ROC: the area of the receiver operating characteristic curve
UCPWI: ultrasound contrast agent plane wave imaging
